# Non-support from the immediate boss is associated with stress and unsafety at work

**DOI:** 10.3389/fpubh.2025.1416609

**Published:** 2025-02-10

**Authors:** Fredrik Iredahl, Elvar Theodorsson, Mike Jones, Tomas Faresjö, Åshild Faresjö

**Affiliations:** ^1^Department of Health, Medicine and Caring Sciences, General Practice, Linköping University, Linköping, Sweden; ^2^Department of Biomedical and Clinical Science, Clinical Chemistry, Linköping University, Linköping, Sweden; ^3^Faculty of Medicine Health and Human Sciences, School of Psychological Sciences, Macquarie University, North Ryde, NSW, Australia; ^4^Department of Health, Medicine and Caring Sciences, Social Medicine and Public Health, Linköping University, Linköping, Sweden

**Keywords:** working conditions, support at work, middle-aged population, job-strain, biological stress, perceived stress

## Abstract

**Background:**

Work-related complaints are often caused by stress and increased mental strain. Support from your immediate boss and colleagues is crucial to buffer against the negative health effects of the psychosocial working environment.

**Aim:**

The aim of this study was to investigate if support from the immediate boss and colleagues was associated with biological stress levels, unsafety at work, and other work-related conditions.

**Methods:**

Data derives from a subsample of the SCAPIS study, a major Swedish prospective population-based study. In this subsample, a total of *N* = 5 058 middle-aged persons (50-64 years) from the general population participated; of these, 68.4% (*N* = 3 462 individuals) provided hair samples. Questionnaires included socio-demographic and self-reports of occupation, stress, and health status. The demand and control questionnaires were used. A biomarker of long-term stress, hair cortisol concentrations (HCC), was also applied.

**Results:**

In this studied cohort, 9.1 % reported a lack of support from their immediate boss, while 90.9% reported that they did get support at work. Significantly more women (*p* < 0.001) reported non-support. Those with support or not did not differ in terms of age, education, civil status, smoking, or ethnicity. Those with non-support reported a higher extent (*p* < 0.001) of lower perceived health. The risk for hypertension and high cholesterol was increased by 28 %, respectively, 13 % being in the non-support group. The main findings were associations between lack of support and feelings of unsafety at work (*p* < 0.001), higher long-term cortisol levels (*p* < 0.009), lack of support from colleagues (*p* < 0.001), and feelings of dejected/sad (*p* < 0.001) and high work pace (*p* = 0.03).

**Conclusion:**

Individuals who did not have the necessary support from their immediate boss and colleagues reported they felt more insecure at work and had higher biological long-term stress. In workplace health promotion, an awareness of the link between social support at work and health could be an important component.

## 1 Introduction

An unhealthy psychosocial work environment can be defined as a job in which the work atmosphere, the colleagues and leadership at work, or a combination of these might negatively affect an employee's health. A poor work environment can lead to serious consequences, especially in the form of chronic stress. Various physical and mental factors can cause chronic stress at work. Chronic stress could have negative effects on many health aspects, like cardiovascular diseases and mental illness (1–4). Chronic stress is also a general risk factor for mental health and well-being in the working population (5, 6). However, one could also consider that a workplace is not necessarily just a source of stress; it could also create a salutogenetic and healthy environment, which would benefit the employees' health (7, 8).

One of the most influential and widely used models in studies on the health effects of psychosocial working conditions and occupational stress is the job-strain or Job-Demand-Control model, initially introduced by Karasek and Theorell (9–11). This model is separated into three aspects regarding workplace qualities: demand, control, and support. The hypothesis behind this model is that work with high demands in combination with high control gives an average strain level and jobs with low demands and low control. The workers suffering from high strain with adverse psychological reactions, such as anxiety and depression, are associated with high demands and low control. High demands, low control, and low support are separate risk factors for anxiety and depression; however, the correlation is even stronger if they are combined (12). Almost all Job Demand-Control model-related studies have principally focused on the combinations of job demands, job control, and social support (12). Another approach to analyze the psychosocial working conditions is the effort-reward model, which has shown adverse health effects yielding high efforts but low rewards (13).

Psychosocial work stress as a potential risk factor for coronary heart disease has been debated. Some researchers have found a causal association, while others found the effect on specific health conditions either negligible or confounded. A major review of evidence from 27 cohort studies in Europe, the USA, and Japan based on data from over 600,000 men and women, suggests that work stressors, such as job strain, are associated with a moderately elevated risk of incident coronary heart disease and stroke. The excess risk for exposed individuals was up to 40% compared to those without such stressors (14). Other studies suggest that job strain could be associated with a consistently increased risk of an incident event of cardiovascular heart disease. Adjustment for conventional risk factors and lifestyle factors together with age, sex, and socioeconomic status, did not substantially change the strength of this association. Prevention of workplace stress can reduce the incidence of cardiovascular diseases, but a more powerful preventive effect is to reduce standard cardiovascular risk factors like smoking (15).

The fear and anxiety of an individual losing their job is another individual stressor (16). Compared to actual job loss, job insecurity is the perception of the employee that his/her job is at risk and could result in employees losing their ability to cope with workplace challenges and threats.

Studies of intermediary pathways from psychosocial job conditions to biomarkers are scarce. Hypertension and high cholesterol are the most investigated cardiovascular risk factors, but the potential associations between psychosocial job conditions and biomarkers of long-term stress are not so well investigated (6).

Social support from jobmates and support from immediate bosses at the workplace are important psychosocial factors in working life. However, the relative impact on health from support at work of the immediate boss has not been extensively studied. Could this type of support at work impact biologically measured stress levels expressed as hair cortisol concentrations (HCC) and perceived stress, and further, if this also affects feelings of unsafety and sadness at work?

## 2 Aim

The study aimed to investigate if a lack of support from the immediate boss and colleagues at the workplace is associated with increased biological and perceived stress levels and feelings of unsafety at work and other adverse working conditions for middle-aged people.

## 3 Material and methods

### 3.1 Study design and participants

Data in the present study constitute a subsample from The Swedish Cardio Pulmonary BioImage Study (SCAPIS). This major prospective national observational study with a randomly selected sample of middle-aged persons (50–64 years) from the general population in Sweden (17–19). The study participants in SCAPIS were recruited from six Swedish university hospitals, and at one of these sites (the Linköping site), additional data collection was initiated, focusing on measuring hair cortisol concentrations (HCC). The total sample at this site was *N* = 5 058 participants; of these, *N* = 3 462 individuals provided hair samples, which were included in this report. Hair samples and thereby also cortisol measurements were unobtainable in 31.5% (*N* = 1 596), mainly due to short hair length. Only a few participants with sufficient hair length were hesitant to provide hair samples. All hair samples were analyzed at the research laboratory of Clinical Chemistry at The University Hospital in Linköping, Sweden.

### 3.2 Questionnaire

The questionnaire includes a comprehensive set of data covering social factors, lifestyle and psychosocial factors. Socioeconomic status was based on educational level and divided into low, medium, and high; smoking was divided into yes, now, or earlier and no. Age was divided into three age groups based on age at study inclusion: 50–54, 55–59, and 60–65. In this studied sample 94.4 % were Swedish and the non-Swedish attendees these were categorized as Nordic, European, or outside Europe. Marital status was divided into single, married/cohabited. The question of economic problems was divided into yes now or earlier and no. A question about self-reported stress was divided into a 2-grade scale of never stressed and quiet or constantly stressed now and the last 5 years. Self-reported health was derived from the SF-36 scale and was dichotomized into good or bad health, and a question about self-reported sleeping habits was also asked. Reports of diagnosed hypertension and elevated plasma cholesterol concentrations were based on self-reports and dichotomized into yes or no. The feeling of dejection was dichotomized into often now or earlier and no. Questions about feeling unsafe at work and forced to change work were divided into yes now or earlier and no. Questions about demand-control-support derived from The Job Demand-Control (JDC) or job-strain models (9, 10).

### 3.3 Hair cortisol concentration analysis

Hair cortisol was extracted and analyzed through a competitive radioimmunoassay (RIA). The hair samples were cut into small pieces. Each sample was put into a 2 mL QiaGenRB sample tube with a 0.5 mm QuiGen stainless steel bead. All samples from the SCAPIS study (4) were analyzed using the same reagents and calibrators to minimize the risk of between-assay bias influencing the study's conclusions. Hair samples over 5–10 mg were required to maintain a total inter-assay coefficient of variation below 8% for hair extraction and measurement of cortisol by the radioimmunoassay. The method is fully described elsewhere (20).

### 3.4 Statistical analysis

The study population was described using Chi-square and ANOVA tests to analyze discrepancies between groups, expressed as median and percentage. Categorical measures are reported as counts and percentages. HCC values and the distribution of HCC were described by median cortisol values and interquartile range (IQR). Hair cortisol was analyzed as an indicator of biological stress where the median is reported. Tests of normality were made with Shapiro-Wilk tests. This showed a non-normal distribution of HCC with a long right-hand tail in the distribution, which is why median and IQR were used. The odds ratio and 95% CI were used as risk estimates.

To correct for potential confounding factors or mediating effects, a multivariate regression analysis based on created models with support from the immediate boss as the dependent variable was performed. Three regression models were made to analyze factors associated with support at work from the immediate boss. Model I (basic model) included only demographic characteristics, HCC, and workplace unsafety. Model II included all covariates from Model I plus work-related factors. The final Model III (or fully adjusted model) included all covariates from Model I and Model II plus health-related factors. A *p* < 0.05 was considered significant in all the analyses. All analyses were performed using SPSS version 28.

## 4 Results

Of the participants in this study who were working (*N* = 2724) 9.1 % reported that they did not get support from their immediate boss, while 90.9% reported that they did get support at work. A fraction of 21.3 % of the participants *N* = 738 in this study reported that they were presently not working.

Comparisons of those who did not get support vs. those who got support from their immediate boss at work revealed that significantly more women (*p* < 0.001) reported non-support. Those with support or not did not differ in terms of age, education, civil status, smoking, or ethnicity. However, those with non-support at work reported a higher and significant (*p* < 0.001) bad health (OR 1.63, 95% CI 1.25–2.13) and everyday stress (*p* < 0.001). They also had tendencies of increased risk for hypertension, high cholesterol, and inferior sleeping habits being in the non-support group. Significantly higher HCC (*p* < 0.001) could also be seen among those who reported bad health. Being a citizen born outside Sweden only showed some increase in HCC concentration (see Table 1).

**Table 1:** Association and odds ratio between non-support and support from the immediate boss at work concerning socio-demographic and health factors (*N* = 3 462).

**Variables**	**Non-support from the immediate boss (*****N*** **=** **247), HCC** = **24.23 (42.79)** ^**a**^		**Support from the immediate boss (*****N*** **=** **2477), HCC**=**23.79 (34.88)** ^**a**^		**OR (95% CI), *p*-value**	**HCC median (IQR)^a^**
	**%**	***n***	**%**	***n***		
**Sex**					0.64 (0.50–0.84) < 0.001	
Female (*N =* 2,180)	53.0	131	63.7	1,578		21.23 (29.32)
Male (*N =* 1,282)	47.0	116	36.3	899		**27.95** (44.97)
**Age-groups**					Na/0.96	
50–54 years (*N =* 1222)	38.1	94	38.1	944		22.94 (31.95)
55–59 years (*N =* 1093)	32.8	81	33.5	831		23.80 (35.58)
60–65 years (*N =* 1147)	29.1	72	28.3	702		**24.56** (38.15)
**Education**					Na/0.37	
Low (*N =* 287)	9.3	23	6.9	171		22.91 (31.21)
Medium (*N =* 1,603)	44.9	111	46.6	1,154		**24.02** (35.43)
High (*N =* 1,485)	45.7	113	46.4	1,149		23.58 (35.08)
**Civil status**					1.01 (0.73–1.39) 0.96	
Single (*N =* 716)	20.6	51	20.5	506		23.37(34.00)
Married/cohabited (*N =* 2,654)	79.4	196	79.5	1,962		23.85 (35.18)
**Ethnicity**					Na/0.61	
Swedish (*N =* 3,258)	93.9	232	94.4	2,339		23.47 (34.57)
Nordic (*N =* 46)	0.8	2	1.3	32		**25.28** (54.85)
European (*N =* 87)	3.6	9	2.4	60		**27.11** (32.10)
Outside Europe (*N =* 71)	1.6	4	1.9	46		**29.83** (44.38)
**Smoking**					0.98 (0.75–1.29) 0.90	
Yes, now or earlier (*N =* 1,393)	39.8	97	40.1	978		23.71(33.57)
No (*N =* 1,933)	60.2	147	59.9	1,458		23.77 (35.67)
**Hypertension**					1.28 (0.93–1.76) 0.13	
Yes (*N =* 656)	22.1	54	18.2	448		**25.45** (42.97)
No (*N =* 2,699)	77.9	190	81.8	2,017		23.50 (34.02)
**High cholesterol**					1.13 (0.72–1.78) 0.58	
Yes (*N =* 314)	9.4	23	8.4	207		**27.86** (53.09)
No (*N =* 3,041)	90.6	221	91.6	2,258		23.50 (33.70)
**Sleeping habits**					1.19 (0.82–1.74) 0.36	
Bad (*N =* 478)	14.2	35	12.2	301		22.23 (34.37)
Good (*N =* 1,627)	85.8	212	87.8	2,173		**24.00** (34.93)
**Self-reported health**					1.63 (1.25–2.13) < 0.001	
Bad (*N =* 1,747)	59.9	148	47.8	1,184		**24.21** (36.91)
Good (*N =* 1,627)	40.1	99	52.2	1,292		23.42 (33.60)

Hair cortisol concentration HCC expressed as a median in the total population is 23.65^*a*^ as an indicator of stress.

The bold mark in the table (a) indicates the HCC median above the general median, IQR measure dispersion, between the 25th percentile and 75th among HCC value in each variable.

There was almost a double risk OR 2.27 (95 % CI 1.65–2.85) of Feeling unsafe at work (39.2% respective 22.9%, *p* < 0.001) being in the non-support group at work compared to those with support. Significant differences were also seen concerning the non-support group compared with the support group among those which was forced to change their work (24% vs. 17.6%, *p* = 0.013, OR 1.47, 95% CI 1.08–2.02), those who reported economic problems (10.5% vs. 6.5%, *p* = 0.02, OR 1.69 (95% CI 1.10–2.61). Non-support and help from colleagues was the strongest indicator for non-support from the immediate boss, 23.8% vs. 1.7%, *p* < 0.001 (OR 17.60, 95% CI 11.55–26.83). Even if the participants had to work fast often a difference was seen 48.6 % vs. 36.4%, *p* < 0.001 (OR 1.65, 95% CI 1.27–2.15). Feeling dejected/sad and always stressed also showed significant differences between the two groups *p* < 0.001 (OR 2.25 95% CI 1.66–3.05) and *p* < 0.001 (OR: 2.22, 95% CI 1.68–2.95).

HCC was also raised among those unsupported at work, always stressed, and rarely received colleague support and help. Being on long-term sick leave was also associated with a small rise in HCC (see Table 2).

**Table 2:** Associations between relevant psychosocial work-related factors concerning support at work from immediate boss.

**Variables**	**Non-support from the immediate boss (*****N*** **=** **247), HCC**= **24.23 (42.79)**^**a**^		**Support from the immediate boss (*****N*** **=** **2,477), HCC** = **23.79 (34.88)**^**a**^		**OR (95% CI), *p*-value**	**HCC median (IQR)a**
	**%**	***n***	**%**	***n***		
**Occupation**					na/0.69	
Full-time work (*N =* 2,421)	83.3	204	84.2	2,069		23.99 (34.91)
Part-time work (*N =* 463)	16.7	41	15.6	382		21.35 (36.07)
Long-term sick leave (*N =* 51)	0.0	0	0.2	5		**25.35** (110.26)
**Feeling unsafety at work**					2.27 (1.65–2.85) < 0.001	
Yes, now or earlier (*N =* 807)	39.2	96	22.9	564		23.50 (34.53)
No (*N =* 2,480)	60.8	149	77.1	1,900		23.86 (36.11)
**Forced to change work**					1.47 (1.08–2.02) 0.013	
Yes, now or earlier (*N =* 632)	24.0	59	17.6	435		23.39 (33.65)
No (*N =* 2,676)	76.0	187	82.4	2,037		23.98 (35.46)
**Economic problems**					1.69 (1.10–2.61) 0.02	
Yes, now or earlier (*N =* 632)	10.5	26	6.5	161		23.33 (28.29)
No (*N =* 2,676)	89.5	221	93.5	2,310		23.83 (35.33)
**Support and help from colleagues**					17.60 (11.55–26.83) < 0.001	
Almost never (*N =* 109)	23.8	58	1.7	43		**27.00** (43.70)
Often (*N =* 2,722)	76.2	186	98.3	2,427		23.62 (35.05)
**Do you have to work fast?**					1.65 (1.27–2.15) < 0.001	
Often (*N =* 1,094)	48.6	120	36.4	901		23.69 (37.85)
Almost never (*N =* 1,803)	51.4	127	63.6	1,576		23.77 (33.52)
**Are you emotionally affected by your work?**					1.37 (1.00–1.91) 0.07	
Often (*N =* 438)	19.1	47	14.8	364		23.78 (37.21)
Almost never (*N =* 2,444)	80.9	199	85.2	2,103		23.91 (35.07)
**Feeling dejected/sad**					2.25 (1.66–3.05) < 0.001	
Often, now or earlier (*N =* 567)	27.2	67	14.2	352		23.50 (28.82)
No (*N =* 2,796)	72.8	179	85.8	2,119		23.86 (35.99)
**Everyday stress**					2.22 (1.68–2.95) < 0.001	
Always (*N =* 693)	34.1	84	18.9	467		**24.05** (33.81)
Seldom or never (*N =* 2,658)	65.9	162	81.1	2,003		23.69 (35.17)

Hair cortisol concentration expressed as the median in the total population is 23.65^*a*^ as an indicator of stress.

The bold mark in table (a) is the HCC median above the general median, IQR measure dispersion, between the 25th percentile and 75th among HCC value in each variable.

In a set of regression models (see Table 3), demographic, health, and working factors were analyzed as co-variants, with support at work from the immediate bosses as the dependent factor. The variables unsafety at work, HCC, work pace, feeling dejected/sad, sex, and colleague support were independently and significantly associated with non-support from the immediate boss at work.

**Table 3:** Three regression models include demographic, health, and working factors as co-variants with support at work from the immediate bosses, as the dependent variable for the whole study population (*N* = 3 462).

**Variables**	**Model 1**			**Model 2**			**Model 3**		
	***B***	***t***	***p*** **-value**	***B***	***t***	***p*** **-value**	***B***	***t***	***p*** **-value**
Constant	–	4.39	< 0.001	–	2.50	0.01	–	1.94	0.05
Unsafety at work	0.095	4.86	< 0.001	0.086	4.19	< 0.001	0.079	3.81	< 0.001
HCC	0.042	2.21	0.03	0.048	2.65	0.008	0.048	2.62	0.009
Everyday stress	0.097	4.96	< 0.001	0.066	3.41	< 0.001	0.033	1.58	0.11
Sex	−0.070	−3.64	< 0.001	−0.047	−2.47	0.02	−0.047	−0.24	0.02
Age-groups	0.005	0.26	0.79	0.010	0.57	0.58	0.013	0.69	0.50
Education	−0.014	−0.73	0.47	−0.001	0.75	0.94	0.002	0.08	0.94
Civil status	−0.010	−0.54	0.59	−0.005	−0.30	0.75	−0.003	−0.44	0.66
Occupation	–		–	0.006	0.32	0.78	0.003	0.17	0.86
Emotionally affected at work	–		–	0.003	0.17	0.86	−0.003	−0.17	0.87
Work pace	–		–	0,.037	1.96	0.05	0.042	2.23	0.03
Support from colleagues	–		–	0.320	17.4	< 0.001	0.324	17.4	< 0.001
Economic problems	–		–	−0.008	−0.40	0.69	−0.015	−0.76	0.45
Forced to change work				−0.009	−0.50	0.65	−0.004	−0.20	0.84
Good/bad health	–		–	-		-	0.028	1.42	0.15
Good/bad sleeping habits	–		–	-		-	−0.001	−0.04	0.96
Dejected/sad	–		–	-		-	0.066	3.25	< 0.001
Smoking							−0.016	−0.81	0.42
Hypertension							0.023	1.19	0.24
High Cholesterol							−0.005	−0.28	0.78

Regression model 1: p < 0.001, df: 7, F= 10.81.

Regression model 2: p < 0.001, df: 13, F= 30.68.

Regression model 3: p < 0.001, df: 19, F= 21.93.

Different clinical and other measures were analyzed for some defined occupational areas, as shown in Supplementary Table S1. The group “workers” showed higher HCC values and more frequently had hypertension and high cholesterol. A higher percentage of the workers did not get any support from the immediate boss. Individuals in service jobs, police/firefighters/bosses, and the military revealed a higher median HCC than the general median in this study population. Among those who were “academics”, support from the immediate boss was higher than in the general population. Often emotionally affected at work was more frequently seen among health care workers, teachers, and those working in children's care, but even among bosses (see Supplementary Table S1).

## 5 Discussion

Social support is regarded as an important factor for health and one of the main dimensions of the classical Demand–control–support model within the field of occupational health (10). The main results of this study reveal that social support from the immediate boss and colleagues at work seems to be an important factor for a healthy workplace. Non-support at work from the immediate bosses, supervisors, or managers was in the final multivariate analysis associated with feeling unsafe at work (*p* < 0.001), higher long-term cortisol levels (*p* < 0.009), lack of support from colleagues (*p* < 0.001) also feelings of dejected/sad (*p* < 0.001) and high work pace (*p* = 0.03), this was more predominant among women (*p* = 0.02). All these factors are related to the internal working environment and the individual.

The results also point to the role of leadership in organizations. Some previous studies have shown a relationship between managers' leadership behaviors and job satisfaction and the performance of employees (21). Leaders with a positive and supportive attitude might motivate employees with a shared vision and activate their inner skills and identities. On the other hand, it has also been seen that abusive leader behavior has negative health consequences for employees. Disinterest or even aggression from immediate bosses, supervisors, or managers could drain employee energies and weaken their psychological resourcefulness, which might result in work withdrawal (22). Accordingly, abusive leadership also affects employees' engagement, ultimately affecting their proactive behavior at work (23, 24). It also puts extra stress on employees, leading to psychological distress and adversely affecting the workplace environment.

The factor of support from colleagues was in this study also significantly associated with non-support from immediate bosses. This illustrates that these two factors are intertwined in many aspects and reflect a positive work environment where social support as a culture is present in the whole organization.

A perception within the Demand–Control–Support model is that social support could act as a buffer against the negative effects of a high job demand on health. Several studies have evaluated this hypothesis to predict worker health, even though it has given some inconclusive results (25). Nevertheless, social support at work seems to be mediated by social support from supervisors and coworkers in different studies of the association of job control with job strain (25).

In this study, the stress indicator HCC was significantly associated with the factor support at work. However, in a previous large Swedish study it was found that the demand control model was not associated with saliva cortisol concentrations (26). However, that study focused on measuring spot-time saliva cortisol levels. In contrast, in the present study, we measured long-term stress exposure through cortisol levels in hair, reflecting the mean cortisol levels for months.

## 6 Strengths and limitations

A major strength of this study is the sample size of middle-aged people and that the participants were randomly selected from the general population. Although the initial purpose of the SCAPIS study was directed toward cardiovascular risk factors, it also included some factors reflecting occupational life. This means that we only have a limited set of relevant factors for measuring psychosocial factors in working life, which gives the analysis some limitations. However, the variables included are well-established and validated.

A general limitation in studies measuring hair cortisol is that individuals with insufficient hair length cannot be included. If insufficient hair length is related to chronic stress, this might be a confounder and impact the results. However, there is no evidence that this might be a confounder in this studied population. A previous analysis of the non-attendees (men without hair samples vs. men with hair samples) in this SCAPIS cohort revealed that they neither differ from the participants concerning socioeconomic background or cardiovascular risk factors (4).” No evidence is at hand that issues and problems in the psychosocial working environment might affect the risk for hair loss.

Ethnicity might be a relevant factor, but in this studied Swedish sample there were relatively few non-Swedish attendees only around 5%. This is mainly explained by the fact that the sample covers older adults in the ages between 50 and 65 years. The Swedish context in this study provides valuable data, but the generalizability of results across contexts in other regions might be limitative. Workplace dynamics may differ significantly in other countries and regions.

## 7 Conclusions

Lack of social support from immediate bosses and colleagues is associated with an increased risk of feeling unsafe at work and higher long-term stress, as measured by cortisol in hair. There are also physical effects such as tendencies to higher blood pressure and cholesterol levels, perceived bad health, feelings of dejected/sad, and sleeping problems.

The prevention of psychosocial stressors in the workplace should emphasize improving a culture where social support is a focus between colleagues, staff, and employees for a good work environment. Promoting awareness of the link between social support at work and health is an important component of workplace health promotion. Social support at work could serve as a salutogenic factor in the workplace.

## Data Availability

The raw data supporting the conclusions of this article will be made available by the authors, without undue reservation.

## References

[1] Steptoe A Kivimaki M. Stress and cardiovascular disease: an update on current knowledge. Annu Rev Public Health (2013) 34:337. 10.1146/annurev-publhealth-031912-114452 23297662

[2] Staufenbiel SM Penninx BW Spijker AT Elzinga BM van Rossum EF. Hair cortisol, stress exposure, and mental health in humans: a systematic review. Psychoneuroendocrinology. (2013) 38:1220. 10.1016/j.psyneuen.2012.11.015 23253896

[3] Vinkers CH Joels M Milaneschi Y Kahn RS Penninx BW Boks MP. Stress exposure across the life span cumulatively increases depression risk and is moderated by neuroticism. Depress Anxiety (2014) 31:737. 10.1002/da.22262 24753162

[4] Faresjö T Stromberg S Jones M Stomby A Karlsson JE Ostgren CJ et al. Elevated levels of cortisol in hair precede acute myocardial infarction. Sci Rep. (2020) 10:22456–224 10.1038/s41598-020-80559-9 33384452 PMC7775435

[5] Goetzel RZ Roemer EC Holingue C Fallin D McClearly K Eaton W. Mental health in the workplace: a call to action proceedings from the mental health in the workplace—public health summit. J Occup Environ Med. (2018) 60:322. 10.1097/JOM.0000000000001271 29280775 PMC5891372

[6] Hassard J Teoh KRH Visockaite G Dewe P Cox T. The cost of work-related stress to society: a systematic review. J Occup Health Psychol. (2018) 23:1. 10.1037/ocp0000069 28358567

[7] Carolan S Harris PR Cavanagh K. Improving employee well-being and effectiveness: systematic review and meta-analysis of webbased psychological interventions delivered in the workplace. J Med Internet Res. (2017) 19:e2 10.2196/jmir.7583 28747293 PMC5550734

[8] Bhui KS Dinos S Stansfeld SA White PD. A synthesis of the evidence for managing stress at work: a review of the reviews reporting on anxiety, depression, and absenteeism. J Environ Public Health. (2012) 2012:5158 10.1155/2012/515874 22496705 PMC3306941

[9] Karasek RA. Job demands, job decision latitude, and mental strain: implications for job redesign. Admin Sci Q. (1979) 24:285–308. 10.2307/239249830315367

[10] Karasek R Thorell T. Healthy Work, Stress, Productivity, and the Reconstruction of Working Life. New York: Basic Books Inc. Publications (1990).

[11] Bjarte S Mykletun A Dahl A Moen B Tell G. Testing the job demand-control-support model with anxiety and depression as outcomes: The Hordaland Health Study. Occup Med-Oxford. (2005) 55:463. 10.1093/occmed/kqi071 15845554

[12] Portoghese I Galletta M Leiter M Finco G d'Aloja E Campagna M. Job demand-control-support latent profiles and their relationships with interpersonal stressors, job burnout, and intrinsic work motivation. Int J Environ Res Public Health. (2020) 17:9430 10.3390/ijerph17249430 33339208 PMC7765581

[13] Siegrist J. Adverse health effects of high-effort/low-reward conditions. Occup Health Psychol. (1996) 1:27. 10.1037//1076-8998.1.1.27 9547031

[14] Kivimäki M Kawachi I. Need for more individual-level meta-analyses in social epidemiology: example of job strain and coronary heart disease. Am J Epidemiol. (2013) 177:1–2. 10.1093/aje/kws407 23144365 PMC3529491

[15] Kivimäki M Nyberg S Batty D Fransson E Heikkilä K Alfredsson L. Job strain as a risk factor for coronary heart disease: a collaborative meta-analysis of individual participant data. Lancet. (2012) 380:1491–7. 10.1016/S0140-6736(12)60994-5 22981903 PMC3486012

[16] Sverke M Hellgren J. The nature of job insecurity: understanding employment uncertainty on the brink of a new millennium. Appl. Psychol. (2002) 51:23. 10.1111/1464-0597.0077z

[17] Bergström G Berglund G Blomberg A Brandberg J Engström G Engvall J et al. The Swedish cardiopulmonary bioimage study: objectives and design. J Intern Med. (2015) 278:645. 10.1111/joim.12384 26096600 PMC4744991

[18] Bergström G Persson M Adiels M Björnson E Bonander C Ahlström H. Prevalence of subclinical coronary artery atherosclerosis in the general population. Circulation. (2021): 144:916. 10.1161/CIRCULATIONAHA.121.055340 34543072 PMC8448414

[19] Östgren CJ Söderberg S Festin K Angerås O Bergström G Blomberg A. Systematic coronary risk evaluation estimated risk and prevalent subclinical atherosclerosis in coronary and carotid arteries: a population-based cohort analysis from the Swedish Cardiopulmonary Bioimage Study. Eur J Prev Cardiol. (2021) 28:250. 10.1177/2047487320909300 33891684

[20] Karlen J Ludvigsson J Frostell A Teodorsson E Faresjo T. Cortisol in hair measured in young adults - a biomarker of major life stressors? BMC Clin Pathol. (2011) 11:12. 10.1186/1472-6890-11-12 22026917 PMC3217842

[21] Koç H. The impact of managers' leadership behaviors on job satisfaction and performance of employees. Afr J Bus Manage. (2011) 5:11836–118 10.5897/AJBM10.122938147025

[22] Chi SCS Liang SG. When do subordinates' emotion-regulation strategies matter? Abusive supervision, subordinates' emotional exhaustion, and work withdrawal. Leadersh Q. (2013) 24:125–1 10.1016/j.leaqua.2012.08.006

[23] Stradovnik K Stare J. Correlation between Machiavellian leadership and emotional exhaustion of employees: case study: Slovenian municipalities. Leader Organ Dev J. (2018) 39:1037–10 10.1108/LODJ-06-2018-0232

[24] Wang CC Hsieh H H Wang Y.D. Abusive supervision and employee engagement and satisfaction: the mediating role of employee silence. Pers Rev. (2020) 49:1845–18 10.1108/PR-04-2019-0147

[25] Blanch A. Social support as a mediator between job control and psychological strain. Soc Sci Med. (2016) 157:148. 10.1016/j.socscimed.2016.04.007 27086104

[26] Alderling M Theorell T de la Torre B Lundberg I. The demand control model and circadian saliva cortisol variations in a Swedish population-based sample (The PART study). BMC Public Health. (2006) 6:2 10.1186/1471-2458-6-288 17129377 PMC1693564

