# Supplementary material for: Non-support from the immediate boss is associated with stress and unsafety at work
Source: Front Public Health. 2025 Feb 10;13:1416609. doi: 10.3389/fpubh.2025.1416609 (PMC11847800; doi:10.3389/fpubh.2025.1416609)
Supplement: Supplementary file 1 [file Table_1.docx]

**Table S1. Hair cortisol concentrations, support at work, hypertension, cholesterol and emotional affected concerning some chosen occupations area for both sexes.**

| **Occupation’s area** | **Median**  **HCC**  **(23.65 pg/mg)^1^** | **Number** | **Hypertension**  **%**  **(19.5 %)^2^** | **High cholesterol %**  **(9.3%)^3^** | **Almost never Support from immediate bosses %**  **(9.1%) ^4^** | **Often**  **emotionally affected at work**  **%**  **(18.9%)^5^** |
| --- | --- | --- | --- | --- | --- | --- |
| Health care workers | 23.20 | 613 | 18.3 | 7.8 | 8.2 | **28.0** |
| Teachers/children’s care | 20.20 | 382 | 20.8 | 9.2 | 5.9 | **17.5** |
| Administrations jobs | 21.45 | 323 | 18.8 | 8.8 | 6.9 | 7.2 |
| Service jobs | **27.19** | 468 | 20.0 | 8.4 | 9.9 | 10.1 |
| Self-employed | **28.52** | 116 | 15.5 | **11.2** | - | 3.3 |
| Civil servants | **24.00** | 479 | 18.0 | 8.6 | 6.9 | 11.0 |
| Workers | 23.39 | 476 | **23.3** | **12.8** | **12.9** | 7.9 |
| Academics | **24.49** | 245 | 13.5 | 7.3 | **12.0** | 6.4 |
| Bosses/managers | **27.60** | 178 | **26.0** | **11.3** | 8.7 | 6.2 |
| Police/firefighters/  military | **27.41** | 41 | 17.1 | **12.2** | 0.0 | 1.2 |

**1.** Total HCC median in SCAPIS. **2.** Total percentage hypertension. **3.** Total percentage with high cholesterol. **4.** Total percentage Almost never Support from bosses. **5**. Often emotionally affected at work

**6**. Bold marks are values above the general median HCC and general percentage.
